# Gut-muscle axis mechanism of exercise prevention of sarcopenia

**DOI:** 10.3389/fnut.2024.1418778

**Published:** 2024-08-16

**Authors:** Tao Li, Danyang Yin, Rengfei Shi

**Affiliations:** School of Health and Exercise, Shanghai University of Sport, Shanghai, China

**Keywords:** sarcopenia, gut microbiota, gut-muscle axis, exercise, SCFAs

## Abstract

Sarcopenia refers to an age-related systemic skeletal muscle disorder, which is characterized by loss of muscle mass and weakening of muscle strength. Gut microbiota can affect skeletal muscle through a variety of mechanisms. Gut microbiota present distinct features among elderly people and sarcopenia patients, including a decrease in microbial diversity, which might be associated with the quality and function of the skeletal muscle. There might be a gut-muscle axis; where gut microbiota and skeletal muscle may affect each other bi-directionally. Skeletal muscle can affect the biodiversity of the gut microbiota, and the latter can, in turn, affect the anabolism of skeletal muscle. This review examines recent studies exploring the relationship between gut microbiota and skeletal muscle, summarizes the effects of exercise on gut microbiota, and discusses the possible mechanisms of the gut-muscle axis.

## 1 Introduction

Sarcopenia is a generalized disease of skeletal muscle consisting of the concurrent combination of reduced muscle mass and muscle strength, the prevalence of which increases with age (1). Sarcopenia can reduce exercise capacity, lead to fall-related injuries such as fractures, reduce the quality of life, and even cause premature death (2). The etiology of sarcopenia is complex, involving neural activity, hormones, immunity, nutrition, physical activity, age, and other factors. The mechanisms of sarcopenia include inflammation, immune senescence, anabolic resistance, and increased oxidative stress (3). Exercise intervention and nutritional supplementation, or a combination of both, are currently considered effective interventions for treating and preventing sarcopenia (4). Additionally, the effects of the gut microbiota on sarcopenia has attracted much attention (5). With aging, skeletal muscle degenerates, while gut microbiota also presents specific changes (6). Gut microbiota changes with specific patterns and transition points with age. In fact, with increasing age, the overall abundance of the gut microbiota decreases while some harmful microbial species increase. The differences between the gut microbiota of adults and elderly people are characterized by a decrease in bacterial diversity, a decline in the number of Bifidobacteria, and an increase in the number of *Clostridium* bacteria, lactic acid bacteria, Enterobacteriaceae, and Enterococcus (7). Whether exercise, as one of the most effective ways to improve and delay muscle loss, can establish a cross-talk between sarcopenia and gut microbiota (also known as the gut-muscle axis) still needs more in-depth discussion.

## 2 Gut microbiota and sarcopenia

### 2.1 Gut microbiota

Gut microbiota refers to the microbial community of bacteria, archaea, viruses, and fungi that live in symbiotic relationships with their hosts in the gastrointestinal tract. A healthy gut microbiota consists of many species, primarily located in the distal part of the gastrointestinal tract (8).

The host and the microorganisms inhabiting it are called “superorganisms,” which can regulate catabolism, synthesize vitamins and amino acids, detoxify foreign substances, regulate immune function, and prevent diseases (9). It also plays a vital role in regulating mitochondrial activity (10) and is part of the central nervous system (11). Gut microbiota may be a determinant of aging due to its regulatory role in human metabolism and immunity (12).

Studies of human gut microbiota have shown that even among healthy people, gut microbiota composition varies significantly between individuals (Human Microbiome Project, 2012). The composition of the gut microbiota is affected by several factors, including diet (13), host genetics (14), delivery mode (15), obesity (16), environment (17), and antibiotic use (18).

### 2.2 Gut microbiota and aging

Gut microbiota have distinct age-related characteristics. Xu quantified the abundance of human gut microbiota through 16S rRNA sequencing and found an aging process in the human gut microbiome, with 35 associated genera proving essential functions through a literature review. Evidence indicated that with age, especially among elderly people older than 90 years, the beneficial effects of the gut microbiota decrease while the effects of inflammation and disease increase (19). Zhang performed 16S rRNA sequencing on the gut microbiota of hospitalized elderly people and found that the abundance of the gut microbiota in frail elderly people was low, which harmed intestinal and overall health (6). Although elderly people have a lower diversity of gut microbiota, different characteristics are found among long-lived elderly people. Tuikhar analyzed the composition of the gut microbiota of centenarians in India and compared it with public data from centenarians in four other countries (India, Italy, Japan, and China). They found that the abundance and biodiversity of the Ruminococcaceae community among centenarians were higher regardless of nationality; the Ruminococcaceae are key symbiotic bacterium of the human gut ecosystem and are a dominant family in healthy ecosystems. The Ruminococcaceae can degrade and convert complex polysaccharides into a variety of nutrients for the host, and their increase may be associated with greater metabolic plasticity and multifunctionality in the gut microbiota of long-lived individuals (20). Bianet analyzed the fecal microbiota of more than 1,000 healthy Chinese individuals, including 198 centenarians in good health. They found that the microbiota composition of centenarians was not significantly different from that of adults aged 30–50 years. However, the abundance of bacteria producing short-chain fatty acids (SCFAs) was higher in centenarians (21).

Among people with physical frailty and sarcopenia, the composition of the gut microbiota has been found to change. It is reported that defects are negatively associated with the abundance and diversity of the gut microbiota in specific categories (22). Picca measured gut microbial levels in frail and sarcopenia elderly people and found an increased abundance of Oscillospira and Ruminococcus microbial taxa and a decreased quantity of Barnesiellaceae and Christensenellaceae compared with the healthy group, confirming that frailty is associated with changes in the composition of the gut microbiota (23). This is consistent with the previous findings that gut microbiota are associated with weakness and biological aging (24).

## 3 Exercise and gut microbiota

### 3.1 Effects of exercise on gut microbiota

At present, the changes in gut microbiota induced by exercise have attracted the attention of many researchers (25). It has been reported that exercise can significantly increase the gut microbiota diversity (26). Exercise regulates metabolism in various ways. It helps to increase energy expenditure, promote glycogen and fatty acid breakdown, improve blood glucose and lipid metabolism, and reduce chronic inflammation, thereby promoting overall health. Studies in humans and rodents have extensively validated the effects of exercise on the immune system (27, 28). Exercise also prevents weakness and cognitive dysfunction by altering gut microbiota (29).

Animal experiments have shown that exercise can regulate gut microbiota significantly. In a mouse model of obesity induced by a high-fat diet, high-intensity exercise ameliorates obesity-induced dysregulation of gut microbiota and maintains microbial diversity (30). In addition, in obese rats, exercise-induced changes in gut microbiota were different from the effects of a healthy diet, suggesting that exercise has an independent impact on gut microbiota. Subsequent studies have found that exercise exerts stronger, more stable, and more influential roles in promoting intestinal mucosal integrity and metabolic function over time (31, 32). In mouse models, moderate-intensity exercise can induce significant changes in the composition of gut microbiota, including increased microbial diversity and levels of critical microbial groups that can promote healthy metabolic activity (33).

In human observational studies, only a few have shown exercise-induced changes in gut microbiota. Bressa found that adult women with an active lifestyle had significantly higher diversity of health-promoting microbiota in stool samples than their sedentary peers (34). According to Clarke, compared with a passive control group of the same size, age, and sex, professional football players had a higher diversity of the gut microbiota, including levels of 22 different microbial groups, which are closely related to protein consumption and creatine kinase (35). Barton also found differences in gut microbiota composition between professional football players and the control group. There are more bacteria-producing SCFAs and more bacteria involved in carbohydrate and amino acid metabolism in the feces of professional football players, resulting in higher concentrations of acetic acid, butyric acid, and propionic acid (36). In a human intervention study, Allen found that exercise-induced changes in the gut microbiota varied based on the obesity status. They conducted 6 weeks of endurance exercise training on both obese and lean sedentary individuals. The study showed that exercise increased fecal short-chain fatty acid concentrations in lean individuals but not in obese individuals. The changes in the gut microbiota were consistent with alterations in genes and bacteria capable of producing SCFAs (37). Moreover, discontinuing exercise training reversed the exercise-induced changes in the gut microbiota, as well as in lean body mass and body fat resulting from the training. Another study by Manuel analyzed the gut microbiota composition of professional cyclists during the Tour de France. It was discovered that the frequency of the consumption of food and sports supplements during the race had a significant impact on the gut microbiota. To enhance athletic performance, future strategies may involve utilizing selective cultures of bacteria that produce SCFAs and targeting specific bacteria (38). However, the above studies only show the differences in the distribution of the gut microbiota among different athletes. More comparisons of healthy gut microbiota among other athletes are needed; the effects of specific sports programs on healthy gut microbiota still need to be investigated.

### 3.2 Exercise intensity plays a significant role in modulating the gut microbiome

The human gut microbiota is significantly influenced by the intensity of physical activity. Generally, long-term exercise of low-to-moderate intensity has positive effects on the gut microbiota, specifically enhancing microbial diversity, elevating the abundance of beneficial bacteria, promoting the production of SCFAs, and strengthening gut barrier function and immune regulation (39). One study found that moderate-intensity exercise significantly impacts the gut microbiota of lean individuals, primarily by enhancing microbial diversity and the production of SCFAs (37). Another study suggested that acute moderate-intensity exercise has positive effects on the gut microbiota of athletes, including increased microbial diversity, a rise in the abundance of probiotic organisms such as Bifidobacteria and Lactobacilli, a decrease in potential pathogenic bacteria, and promotion of the production of SCFAs, thus enhancing gut barrier function and anti-inflammatory effects (40).

In contrast, high-intensity exercise might have different impacts on gut microbiota. During high-intensity workouts, the body’s energy is chiefly spent on sustaining muscular activity and other physiological functions, which might reduce the available energy for gut microbiota, thereby affecting their growth and metabolic activities (41). Additionally, high-intensity exercise might induce intestinal and systemic inflammatory responses, thereby increasing the levels of inflammatory markers, such as C-reactive protein (CRP) and tumor necrosis factor-α (TNF-α). These persistent inflammatory responses could disrupt the stability and diversity of the gut microbiota, leading to a decrease in beneficial bacteria and an increase in potential pathogenic bacteria (39). Further research has indicated that high-intensity exercise could increase intestinal permeability, causing damage to intestinal epithelial cells and heightening the potential risk of leaky gut syndrome (42). Some specific changes, e.g., a decrease in beneficial bacteria (such as Lactobacilli and Bifidobacteria) and an increase in potential pathogenic bacteria (such as Clostridia), are common in individuals who engage regularly in high-intensity and long-duration workouts. The gut microbiota characteristics of these athletes show higher counts of bacteria involved in the inflammatory process (43). Therefore, workout intensity is a crucial factor affecting the state of the gut microbiota.

### 3.3 Possible mechanisms of how exercise affects gut microbiota

**Figure 1:**
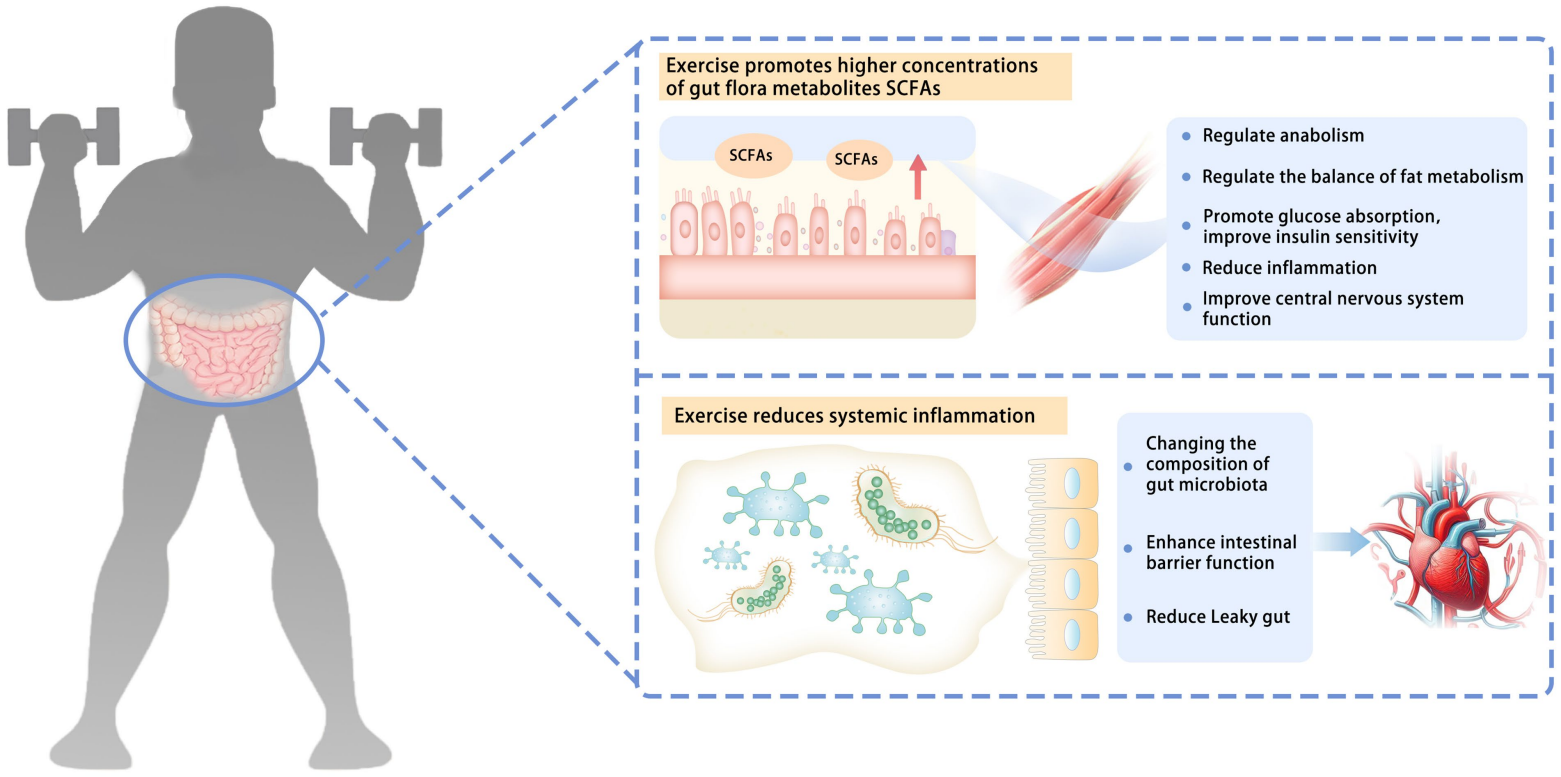
Possible mechanisms of how exercise affects gut microbiota. The effects of exercise on human gut microbiota may involve a variety of mechanisms, such as influencing the production of intestinal microbial metabolites and regulating immune response. Exercise promotes higher concentrations of gut flora metabolites, SCFAs. SCFAs can be absorbed by skeletal muscle through systemic circulation. They can regulate anabolism, regulate the balance of fat metabolism, promote glucose absorption, improve insulin sensitivity, reduce inflammation, and improve the function of the central nervous system. Exercise reduces systemic inflammation. Changing the composition of the gut microbiota enhances intestinal barrier function and reduces the leaky gut. SCFAs, short-chain fatty acids; leaky gut, gut microbiota from the intestinal cavity to the blood circulation.

The effects of exercise on human gut microbiota may involve multiple levels, such as regulating immune response (44) and influencing the production of intestinal microbial metabolites, including SCFAs (8, 45) (Figure 1).

#### 3.3.1 Gut microbiota metabolites of SCFAs

The effect of exercise on gut microbiota mainly manifests as an increase in SCFA concentration. SCFAs, fatty acids of 2–6 carbon atoms, are primarily produced by the fermentation of indigestible sugars, such as dietary fiber and oligosaccharides, by beneficial bacteria, such as *Lactobacillus* and *Bifidobacterium* in the colon. SCFAs can be absorbed by skeletal muscle through systemic circulation. They can regulate metabolic synthesis processes, such as activating AMPK and mTOR signaling pathways and enhancing mitochondrial biogenesis (46), by inhibiting histone deacetylases (HDACs) and activating transcription factors like PPARγ. SCFAs can promote the expression of genes related to fatty acid synthesis and storage, influence the AMPK signaling pathway, and regulate the activity of fatty acid synthase (FAS), balancing the body’s fatty acid synthesis and oxidation processes (47). By inducing the translocation of glucose transporter 4 (GLUT4) and regulating the phosphorylation status of insulin receptor substrate (IRS), SCFAs can enhance insulin signal transduction, activate the phosphatidylinositol 3-kinase (PI3K) and protein kinase B (Akt) pathways to promote glucose absorption, and increase insulin sensitivity (48). Through various mechanisms, such as inhibiting the NF-κB signaling pathway, activating G protein-coupled receptors, adjusting the acetylation state of histones, regulating immune cell function, and inhibiting pro-inflammatory metabolic pathways, SCFAs may potentially reduce inflammation (49). SCFAs can alter the permeability of the blood–brain barrier, influencing metabolism and signal transmission within the brain, and improving central nervous system functions (50). In addition, butyrate among SCFAs can enhance ATP generation and muscle fiber metabolism efficiency (51).

Physical exercise may stimulate the production of SCFAs. Research by Allen reveals that exercise has the potential to alter the composition and functionality of human gut microbiota. Among lean participants, exercise significantly increases the concentration of SCFAs in fecal matter (37). Furthermore, exercise-induced intestinal hypoxia involves intricate mechanisms impacting gut microbiota and their metabolic byproducts. The rerouting of blood during physical exercise prioritizes supply to muscles and other active organs, resulting in decreased blood flow and oxygen delivery to the intestines. This oxygen-depleted environment in the gut increases the growth of anaerobic bacteria, whose compositional shifts subsequently influence the formation of metabolic products, such as SCFAs, within the gut. A study by Huang observed that acute exercise-induced hypoxia increased changes in the gut microbiota of mice. This promoted the proliferation of anaerobic bacteria such as *Akkermansia* and *Bacteroidetes* genera, accompanied by an increase in the production of short-chain fatty acids. These changes ultimately improved the endurance performance of the mice (52).

The mechanism by which exercise promotes increased SCFA concentration is still being determined. The possible mechanism is that changes in colonic oxygen saturation or pH may encourage the mixing of intestinal contents and the fermentation of dietary fiber through endogenous metabolite input (e.g., lactic acid), thereby increasing the production of SCFAs (53).

Exercise induces an increase in SCFAs in feces, which may also be due to an increased ability of the gut microbiota to produce SCFAs. In the process of SCFA production, butyryl-CoA is a critical enzyme for butyric acid production (54). Exercise may induce the expression of genes for these vital enzymes, which may partially explain the increase in SCFAs in feces.

#### 3.3.2 Systemic inflammation

Another potential mechanism involves changes in systemic inflammation levels. Animal experiments have found that exercise can mediate alterations in metabolism and attenuate colitis in mice (55). Gut microbiota plays a role in the intestinal immune response by mediating the transfer of gut microbiota from the intestinal cavity to the blood circulation, a phenomenon known as leaky gut. Exercise can enhance intestinal barrier function and reduce inflammation by regulating the composition of the gut microbiota (56). Therefore, exercise may help reduce inflammation.

## 4 The effects of gut microbiota on skeletal muscle

In recent years, it has been reported that the gut microbiota is involved in skeletal muscle metabolism (57). The composition of the gut microbiota may affect the quality, structure, and function of skeletal muscle during aging, thus influencing the development of sarcopenia.

### 4.1 Muscle function

The gut microbiota significantly affects skeletal muscle mass and function in mice. Research has shown that germ-free mice lacking gut microbiota experience skeletal muscle atrophy, reduced expression of insulin-like growth factor 1, and decreased transcription of genes related to muscle growth and mitochondrial function. However, transferring gut microbiota from pathogen-free mice to germ-free mice helps improve these conditions. These findings highlight the crucial role of the gut microbiota in regulating skeletal muscle function (58). In another study, treatment with the antibiotic metronidazole led to dysregulation of the gut flora in mice, resulting in reduced hindlimb muscle weight, smaller fibers in the tibialis anterior muscles, and upregulation of genes associated with neurogenic atrophy of skeletal muscle, including Hdac4, myogenin, MuRF1, and atrogin1, which contribute to skeletal muscle atrophy (59). Additionally, research showed that 6 weeks of supplementation with *Lactobacillus plantarum* TWK10 in mice enhanced glucose utilization by increasing the number of gastrocnemius type I muscle fibers, resulting in increased endurance exercise time (60).

Human studies have also found that gut microbiota composition is related to skeletal muscle function in elderly people. For instance, when elderly people are transferred to care facilities, their gut microbiota changes, affecting bone and body composition, which can lead to muscle loss, osteoporosis, obesity, and an increased risk of fractures (61).

### 4.2 Muscle mass

Animal experiments have shown that changes in gut microbiota can affect skeletal muscle mass. The BaF3 mouse model is a typical model of leukemia that exhibits muscle atrophy, fat loss, anorexia, and inflammatory responses at later stages. Significant changes in the gut microbiota of these models, particularly in the *Lactobacillus* populations, were found in this study. The balance of *Lactobacillus* populations could be restored by supplementing BaF3 mice with *Lactobacillus reuteri* 100-23 and *Lactobacillus gasseri* 311,476, and the expression of muscle atrophy markers (Atrogin-1, MuRF1, LC3, and histone L) was reduced in the gastrocnemius and tibialis muscles. Additionally, the levels of serum factors related to chronic inflammation (e.g., IL-6, MCP-1) were reduced. These findings suggest that the gut microbiota, especially *Lactobacillus* species, can modulate the body’s immune-inflammatory response and inhibit muscle atrophy (62). Chen has shown that long-term exposure to *L. plantarum* in mice may increase energy absorption and muscle mass (60).

Prebiotics may affect skeletal muscle through gut microbiota by promoting the proliferation of beneficial bacteria (63). Cani found that prebiotic supplements reduced lipopolysaccharide (LPS) levels and inflammation, promoting increased skeletal muscle mass in obese mice (64). Varian found a significant improvement in muscle mass after treating mice with muscle atrophy using a probiotic mixture containing *L. reuteri* (65). Probiotics and prebiotics may promote skeletal muscle anabolism by improving gut microbiota composition, reducing intestinal endotoxin concentrations, and increasing insulin sensitivity (66).

### 4.3 Muscle composition

Increasing age leads to increased infiltration of adipose tissue, altering the composition of skeletal muscle, which associated with a decrease in motor skills (67). Remarkably, resistance training can mitigate this accumulated fat by increasing the size of muscle fibers; in contrast, aerobic exercises contribute to fat burning by boosting the overall metabolic rate (68). Regular physical activity further enhances metabolic health and helps prevent the accumulation of fat in muscle tissue (69). Importantly, gut microbiota regulates inflammation and fat metabolism, particularly through the production of substances such as SCFAs. This aids in improving insulin sensitivity, which subsequently helps reduce the accumulation of fat in muscles (70).

In animal models, changes in gut microbiota also alter the composition of skeletal muscle fibers (62). Studies have shown that a high-fat diet induces acute changes in the gut microbiota of 3-month-old rats and significantly affects its overall composition within 28 days. This leads to increased circulating inflammatory cytokines and intramuscular fat in skeletal muscle. Furthermore, a high-fat diet alters intestinal barrier function by increasing pro-inflammatory cytokines, such as TNF-α and IL-6, modulating metabolic pathways of SCFAs, affecting insulin signaling pathways, altering intestinal barrier function and skeletal muscle composition and function, and possibly causing shifts in myofiber type. Together, these mechanisms reveal the complex relationship between the gut microbiota and skeletal muscle composition (71). A study on 10-month-old pigs found that energy metabolism-related gut bacteria were significantly associated with intramuscular fat content through 16S rRNA gene sequencing, validating the critical role of the gut microbiota in regulating muscle composition (72).

## 5 Possible mechanisms of the gut-muscle axis

**Figure 2:**
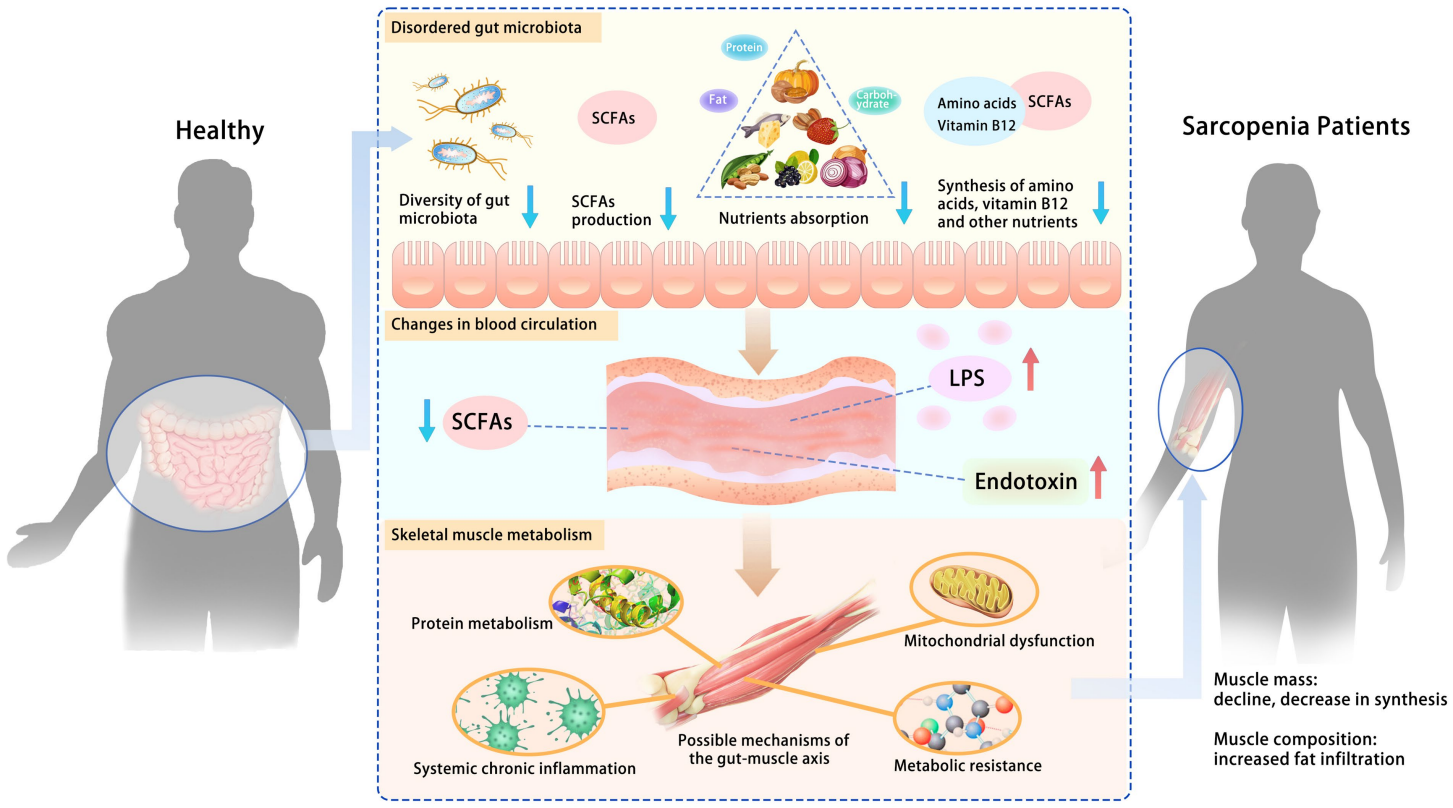
Possible mechanisms of the gut-muscle axis. Disturbances in gut microbiota increase intestinal permeability, leading to changes in blood circulation and further affecting skeletal muscle metabolism. The role of the gut microbiota in regulating skeletal muscle has been confirmed. Some studies have suggested a “gut-muscle axis” and proposed possible mechanisms by which gut microbiota affects skeletal muscle mass and function, including protein metabolism, systemic chronic inflammation, metabolic resistance, and mitochondrial dysfunction. SCFAs, short-chain fatty acids; LPS, lipopolysaccharide.

Animal models and human studies have confirmed the role of the gut microbiota in regulating skeletal muscle, but its systemic mechanisms still need to be fully understood. Some studies have suggested that there is a “gut-muscle axis” and proposed possible mechanisms (73) by which gut microbiota affects skeletal muscle mass and function, including protein metabolism, systemic chronic inflammation, metabolic resistance (reduced or diminished responsiveness of the organism in metabolic regulation), and mitochondrial dysfunction (Figure 2).

### 5.1 Metabolites of the gut microbiota

Metabolites produced by the gut microbiota can be absorbed by the intestinal mucosa and affect skeletal muscle quality (74). Some of these metabolites are nutrients, such as folic acid (75), riboflavin (76), vitamin B12 (75), betaine (77), and some amino acids (78). These nutrients affect skeletal muscle function, from promoting DNA synthesis and repair to mediating insulin growth factor 1, which stimulates anabolism and cell proliferation (73). Healthy gut microbiota can produce these nutrients and improve the bioavailability of amino acids. In contrast, dysregulation of the gut microbiota may lead to reduced production of these nutrients and decreased absorption of amino acids, negatively affecting skeletal muscle anabolism (78).

In addition, gut microbial metabolites represented by SCFAs (such as acetate, propionate, and butyrate) can act as endocrine mediators and influence the metabolism and function of skeletal muscles (79). They can improve glucose homeostasis and insulin sensitivity, regulate inflammation and satiety, and stimulate catabolism in adipose tissue and anabolism in skeletal muscle cells. Dysregulation of the gut microbiota is usually characterized by the reduced production of SCFAs, resulting in reduced anabolic or anti-catabolic action of skeletal muscle (73).

### 5.2 Protein anabolism

Gut microbiota are involved in the metabolism and absorption of amino acids. They can improve the bioavailability of amino acids by utilizing several amino acids from nutrient and endogenous proteins, thus affecting the synthesis and decomposition of muscle proteins (80).

In addition, gut microbiota can synthesize some essential amino acids, such as tryptophan (78), which may stimulate the insulin-like growth factor 1/p70S6K/mTOR pathway in muscle cells and promote the expression of muscle fiber synthesis genes (81).

The composition of the gut microbiota can also influence muscle mass, anabolism, and host function by producing mediators, such as SCFAs (73). They can increase the production of ATP by regulating the protein regulation pathway, affect protein deposition, and stimulate the glucose absorption of skeletal muscle by regulating the balance of synthesis and decomposition throughout the body (82).

### 5.3 Inflammation and gut barrier function

The aging process can seriously affect the structure of the human gut microbiota and the balance and inflammatory state of the host immune system. Changes in the gut microbiota can alter the inflammatory state of individuals.

Healthy gut microbiota regulates immune homeostasis, maintains the balance between pro-inflammatory and anti-inflammatory responses, and suppresses chronic inflammation (73). Meanwhile, it can induce host reactions in the intestinal mucosa, strengthen the intestinal barrier function, and play an immunomodulatory role inside and outside the intestinal tract (83). SCFAs produced by the gut microbiota can exert anti-inflammatory effects by reducing the secretion of pro-inflammatory cytokines and chemokines as well as the infiltration of local macrophages. Butyric acid, one of the SCFAs can induce the secretion of interleukin-10, retinoic acid, and tumor growth factor-β (84), stimulate the production of anti-inflammatory T cells (85), and suppress inflammation.

Another mechanism by which gut microbiota regulates inflammation is maintaining intestinal barrier function, regulating LPS, and promoting the absorption of inflammatory bacterial endotoxin (86). Butyrate also plays a vital role in intestinal barrier function by strengthening the tight junctions of epithelial cells, thus preventing endotoxin translocation and reducing inflammation (87).

### 5.4 Mitochondrial dysfunction

Mitochondria is the energy factory of the cell. Mitochondrial function, especially ATP production and the efficiency of the respiratory chain, decreases with age. In skeletal muscle, primary aging results in a lack of mitochondrial energy and a loss of muscle mass (88). Downregulation of genes of the mitochondrial energy metabolism pathway (e.g., the tricarboxylic acid cycle and oxidative phosphorylation) causes mitochondrial dysfunction and progressive loss of muscle mass and function, leading to sarcopenia (89). The inducible inflammatory factors in sarcopenia may be the free mtDNA of nucleoid or oxidized cells extruded from the damaged mitochondria, which can activate the innate immune system of the body and induce the production of inflammatory mediators. The release of inflammatory mediators can sustain a vicious cycle within the muscle cells, leading to further mitochondrial damage and, eventually, sarcopenia (90).

There is a regulatory relationship between gut microbiota and mitochondria, and interrupting this relationship may lead to mitochondrial dysfunction (91). The gut microbiota can regulate key mitochondrial transcriptional activators, transcription factors, and enzymes such as PGC-1α, SIRT1, and AMPK genes (10).

In addition, SCFAs are recognized as mediators underlying the effects of the gut microbiota on skeletal muscle, and their main targets are mitochondria of skeletal muscle cells, suggesting a close relationship between gut microbiota and mitochondrial function. SCFAs and secondary bile acids can promote mitochondrial energy production and regulate reactive oxygen species (ROS) by weakening TNF-α-mediated immune responses and inflammatory bodies such as NLRP3. Additionally, SCFAs, such as butyric acid and acetic acid, can directly or indirectly affect mitochondrial energy metabolism through a wide range of transcription factors (10).

### 5.5 Insulin resistance and metabolism resistance

Elderly people have anabolic resistance and require more protein intake to achieve the same myoprotein synthesis as younger adults (92). Gut microbiota participate directly or indirectly in the hypothesized mechanisms of anabolic resistance in elderly people and may facilitate complex interactions among these hypothesized mechanisms (93). Thus, a healthy gut microbiota may reduce metabolic resistance.

Disordered gut microbiota and altered intestinal barriers may reduce SCFAs and secondary bile acids but increase the absorption of LPS and branched-chain amino acids (BCAAs). All of these changes can lead to insulin resistance (85). SCFAs are critical mediators of mitochondrial energy metabolism, acting as ligands for free fatty acid receptors 2 and 3 and regulating glucose and fatty acid metabolism. They can enhance glucose tolerance, promote insulin sensitivity, and play a key role in regulating glucose uptake and metabolism (73). Secondary bile acids can activate the secretion of glucagon-like peptide-1, thereby reducing insulin resistance and maintaining glucose homeostasis (94). LPS can activate Toll-like receptor 4 signaling, inducing insulin resistance, inflammation, and obesity (85). Circulating BCAA levels may independently increase the risk of insulin resistance and type 2 diabetes (95).

## 6 Conclusion and limitations

In summary, the impact of exercise on the gut microbiota is multifaceted, leading to notable changes such as increased abundance and the production of SCFAs. Concurrently, the gut microbiota exerts a profound influence on skeletal muscle quality and function through various mechanisms, such as the regulation of protein metabolism, mitigation of insulin resistance, improvement of mitochondrial function, and attenuation of inflammation. As individuals grow older, people may experience sarcopenia. This aging-related process can significantly alter intestinal physiology, particularly the integrity of the intestinal mucosal barrier. Consequently, shifts in gut microbiota composition may arise as a consequence of sarcopenia rather than serving as its primary cause. Moreover, sarcopenia frequently coincides with inadequate nutrition and diminished exercise capacity. Given that both diet and physical activity strongly influence gut microbiota composition, it is plausible that lifestyle factors contribute to sarcopenia rather than solely attributing it to dysbiosis of the gut microbiota.

However, existing research on the relationship between gut microbiota and sarcopenia is limited. Many studies have been conducted on small cohorts, lacking comprehensive assessments of elderly populations. Furthermore, the majority of data concerning the interplay between gut microbiota and muscle mass/function are derived from animal models, with human studies primarily being observational in nature. Therefore, the precise role of the gut microbiota in the development and progression of sarcopenia warrants further investigation and a broader scope of research methodologies.

## Author contributions

TL: Investigation, Methodology, Writing – original draft, Writing – review & editing. DY: Methodology, Writing – review & editing, Writing – original draft. RS: Funding acquisition, Methodology, Supervision, Writing – review & editing, Writing – original draft.
